# Dual-use virulence factors of the opportunistic pathogen *Chromobacterium haemolyticum* mediate hemolysis and colonization

**DOI:** 10.1128/mbio.03605-24

**Published:** 2025-04-03

**Authors:** Leo Dumjahn, Philipp Wein, Evelyn M. Molloy, Kirstin Scherlach, Felix Trottmann, Philippe R. Meisinger, Louise M. Judd, Sacha J. Pidot, Timothy P. Stinear, Ingrid Richter, Christian Hertweck

**Affiliations:** 1 Department of Biomolecular Chemistry, Leibniz Institute for Natural Product Research and Infection Biology (Leibniz-HKI) 28406, Jena, Germany; 2 Department of Microbiology and Immunology, Doherty Institute, University of Melbourne, Melbourne, Australia; 3 Institute of Microbiology, Faculty of Biological Sciences, Friedrich Schiller University Jena, Jena, Germany; 4 Cluster of Excellence Balance of the Microverse, Friedrich Schiller University Jena 9378, Jena, Germany; Max Planck Institute for Marine Microbiology, Bremen, Germany

**Keywords:** jagaricins, nonribosomal peptide, cyclic lipopeptides, hemolysis, biofilm, swarming

## Abstract

*Chromobacterium haemolyticum* is an environmental bacterium that can cause severe and fatal opportunistic infections in humans and animals. Although *C. haemolyticum* is characterized by its strong β-hemolytic activity, the molecular basis of this phenotype has remained elusive over the more than 15 years since the species was first described. Herein, we report a family of cyclic lipodepsipeptides, the jagaricins, that are responsible for the potent hemolytic activity of *C. haemolyticum*. Comparative genomics of *C. haemolyticum* strains revealed a completely conserved gene locus (*hml*) encoding a nonribosomal peptide synthetase. Metabolic profiling of *C. haemolyticum* DSM 19808 identified a suite of cyclic lipodepsipeptides as the products, with the three main congeners (jagaricin A–C) being elucidated by a combination of tandem mass spectrometry, chemical derivatization, and nuclear magnetic resonance spectroscopy. Significantly, a *C. haemolyticum hml* deletion mutant is devoid of hemolytic activity. Moreover, purified jagaricins are hemolytic at low micromolar concentrations in an erythrocyte lysis assay. Further bioassays demonstrated that the cyclic lipodepsipeptides are crucial for the biofilm-forming and swarming behavior of *C. haemolyticum*. Matrix-assisted laser desorption ionization mass spectrometry imaging showed that primarily jagaricin B and C are involved in these processes *in vitro*. Our data shed light on the bioactivities of jagaricins, specialized metabolites that likely contribute to both successful niche colonization and the virulence potential of *C. haemolyticum*.

**IMPORTANCE:**

Despite the rising incidence of *Chromobacterium haemolyticum* as a serious opportunistic pathogen, there is limited information on whether the competitive traits that ensure its survival in its freshwater niche also influence host infection. We reveal that *C. haemolyticum* produces specialized metabolites that not only cause its pronounced hemolytic phenotype but are also crucial for biofilm formation and swarming motility. These results exemplify a case of coincidental evolution, wherein the selective pressures encountered in a primary environmental niche drive the evolution of a trait impacting virulence. This knowledge provides a foundation for the development of antivirulence therapies against the emerging pathogen *C. haemolyticum*.

## INTRODUCTION

Environmental microbes interact with a broad range of microorganisms through cooperation and competition (1, 2). Survival likely requires numerous adaptations to withstand the constant barrage of both biotic and abiotic stressors. Occasionally, an adaptation emerges that may incidentally act as a virulence factor when an environmental microbe encounters a susceptible host (3–5). In contrast to host-acquired pathogens, environmentally acquired pathogens seemingly do not require a host stage in their life cycles and display virulence traits shaped by selection in a distinct ecological niche (5). For example, the mechanisms that protect *Legionella* and *Salmonella* strains from protozoan soil predators also aid their survival within human macrophages (6, 7). The production of Shiga toxins by *Escherichia coli* O157:H7 to fend off grazing protozoa (*Tetrahymena pyriformis*) is thought to aid in the colonization of humans (8). The opportunistic fungal pathogen *Rhizopus microsporus* is likewise endowed with resistance to both amoebae and human macrophages by its bacterial endosymbiont (*Ralstonia pickettii*), illustrating how an environmentally evolved tripartite interaction can lead to enhanced virulence (9).

A prime example of a bacterium equipped with a variety of competitive traits for survival in its ecological niche is *Chromobacterium haemolyticum* (10–13). First isolated from sputum (10), numerous strains have subsequently been isolated from freshwater sources (11–13). This facultatively anaerobic, gram-negative bacterium is known for its pronounced antibacterial effect against *Salmonella*, *E. coli*, *Listeria monocytogenes*, and *Staphylococcus aureus* (14). In addition, *C. haemolyticum* displays mosquitocidal activity and inhibits the growth of *Plasmodium falciparum* through the production of cyanide and the histone deacetylase inhibitor romidepsin, respectively (13, 15). In recent years, there has been an alarming increase in the number of reports of severe opportunistic infections in animals (16) and humans (17–24) caused by *C. haemolyticum*. In many cases, *C. haemolyticum* infections are associated with exposure to freshwater, indicating a potential transmission route from the natural habitat to the host (16, 18–21, 24).

*C. haemolyticum* can infect different organs and body parts, causing proctocolitis (17), pneumonia (18, 19), meningitis (20), or soft tissue infections (21, 22) that can lead to bacteremia (21, 23) and eventually, sepsis (23, 24). These infections are difficult to treat due to a combination of resistance of *C. haemolyticum* to antibiotics (10, 25, 26) and its frequent misidentification as *Chromobacterium violaceum*, the more common representative of the *Chromobacterium* genus, potentially resulting in ineffective treatment (18–20, 23). Perhaps the most striking characteristic of *C. haemolyticum* is its pronounced β-hemolytic activity, which gives the species its name (10). Hemolysis can serve as a key virulence factor in mammalian infections, particularly given its association with sepsis (27). Although various virulence factors of *C. haemolyticum* have been linked to human (28, 29) and animal (13–15) diseases, the hemolytic phenotype of *C. haemolyticum* has not yet been investigated in this regard, and its molecular basis remains unknown.

Here, we report the analysis of a human pathogenic *C. haemolyticum* strain by comparative genomics and metabolic profiling. We characterize the jagaricins, a family of cyclic lipodepsipeptides produced by a nonribosomal peptide synthetase (NRPS), which we find are responsible for *in vitro* hemolysis. Additionally, we demonstrate a critical role for jagaricins in *C. haemolyticum* swarming and biofilm formation. Taken together, our findings present a rare case of dual-use specialized metabolites that can both advance niche colonization and presumably augment the virulence of an environmentally acquired pathogen.

## RESULTS

### Genome mining reveals a cryptic NRPS biosynthetic gene cluster that is conserved in *C. haemolyticum* strains

**Fig 1:**
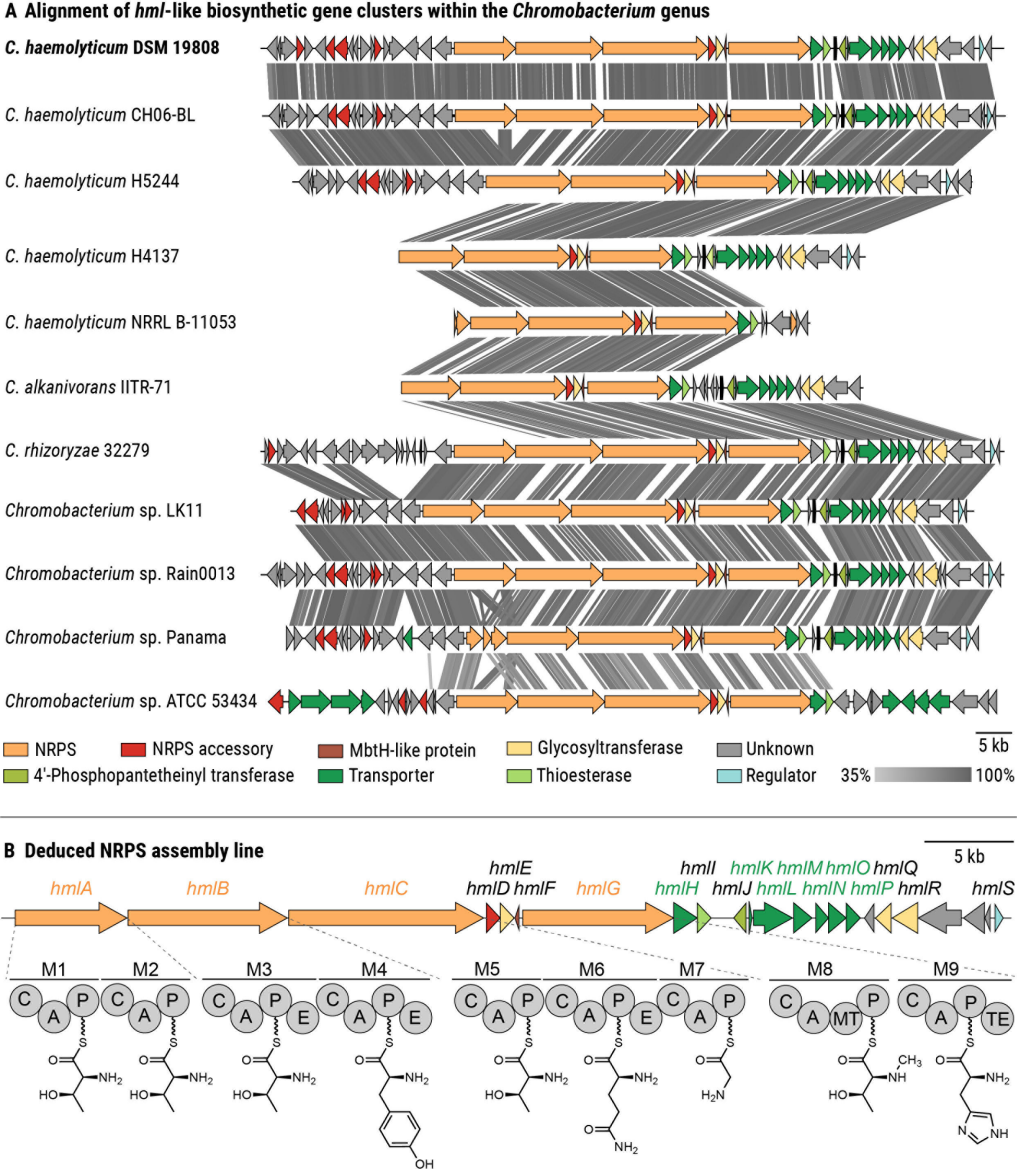
Identification of a cryptic NRPS-encoding biosynthetic gene cluster in the genome of the opportunistic pathogen *C. haemolyticum*. (A) Alignment of *hml*-like BGCs within the *Chromobacterium* genus. Colored arrows represent open reading frames, with predicted functions of the products indicated in the key. The gray bar indicates the similarity of homologous regions, which are connected by gray lines. (B) Depiction of the deduced NRPS assembly line of the predicted *hml* BGC identified in the genome of *C. haemolyticum* (GenBank accession: PQ303793). It should be noted that the order of amino acid incorporation is depicted as bioinformatically predicted with one exception. Based on the elucidated structures of the cognate metabolites (Fig. 3), the last amino acid is given as histidine rather than leucine. C, condensation domain; A, adenylation domain; P, peptidyl-carrier protein domain; E, epimerization domain; MT, *N*-methyltransferase domain; TE, thioesterase domain; M1–M9, modules 1–9.

Because hemolysis can be induced by structurally diverse molecules, ranging from small peptidic natural products to large enzymes, we used a comparative genomics approach to try to pinpoint the biosynthetic origin of hemolysins within the *Chromobacterium* genus. We noted a cryptic biosynthetic gene cluster (BGC) that is conserved among all *C. haemolyticum* strains, including the sputum isolate that prompted the recognition of the species, i.e. *C. haemolyticum* DSM 19808 (Fig. 1A). This putative BGC was named *hml* (76.8 kb, Fig. 1B) because of its universal presence in *C. haemolyticum* strains. In the case of *C. haemolyticum* DSM 19808, the *hml* BGC was found on a contig edge of the publicly available whole genome sequence (GenBank accession of whole genome sequence: ASM71188v1), necessitating the re-sequencing of the region (GenBank accession of *hml* BGC: PQ303793). Besides *C. haemolyticum*, only the phylogenetically closely related *Chromobacterium rhizoryzae* (30) and *Chromobacterium alkanivorans* (31), as well as four *Chromobacterium* sp. isolates, harbor the *hml* BGC (Fig. 1A). This putative BGC has not yet been associated with a natural product but putatively codes for the biosynthesis of a nonribosomal peptide.

To determine whether the *hml* BGC is unique to *Chromobacterium* spp., we constructed a genome neighborhood network using the ESI-Genome Neighborhood Tool and found similar BGCs in several cyanobacteria (e.g., *Anabaena* spp., *Cylindrospermopsis raciborskii*, and *Planktothrix sertra*) (Fig. S1). These BGCs encode the biosynthetic enzymes for the production of hassallidins, which are cyclic lipopeptides with antifungal properties (32–35). Moreover, we noted similarities in terms of gene assignments between the *hml* BGC and the *jag* BGC from the mushroom soft rot pathogen *Janthinobacterium agaricidamnosum* (Fig. S1). Intriguingly, the corresponding secondary metabolite, jagaricin, is a cyclic lipopeptide with antifungal and hemolytic activities (36, 37).

Bioinformatic analysis of the *C. haemolyticum hml* BGC (38) revealed that the predicted NRPS is encoded by four genes (*hmlA–C* and *hmlG*), comprising nine modules responsible for chain elongation and an off-loading thioesterase domain (Fig. 1B). The amino acid sequence of the nonribosomal peptide backbone was predicted using the Stachelhaus codes of the adenylation (A) domains, resulting in the tentative nonapeptide Thr–Thr–d-Thr–d-Tyr–Thr–d-Gln–Gly–*N*-Me-Thr–Leu (Fig. 1B; Table S1). Noting that threonine residues can be dehydrated to the corresponding dehydrobutyrine residue via specialized condensation (C) domains (39), the peptide sequence resembles the core sequence found in both hassallidins and jagaricin, differing only in the predicted leucine at the C-terminal position (Table S1). Module 1 contains an additional condensation domain that could catalyze an *N-*terminal acylation, e.g., with a fatty acid. Due to the epimerization (E) domains present in modules 3, 4, and 6, the corresponding Thr, Tyr, and Gln residues are predicted to be isomerized to the d-enantiomers. Module 8 contains an *N*-methyltransferase domain that might catalyze the *N*-methylation of the threonine incorporated at the eighth position. Several additional biosynthetic enzymes are encoded in the BGC: a type-II-thioesterase (*hmlI*) that could be responsible for restoring NRPS activity when a module is misloaded (40, 41) and three glycosyl transferases (*hmlE, hmlQ*, and *hmlR*) that might glycosylate the NRPS product, thus forming a glycopeptide. *N*-methylation of the eighth residue, as well as the incorporation of sugar moieties, are modifications that are also present in the hassallidins (35). Based on comparative genomics and bioinformatic prediction, our data indicate that *C. haemolyticum* DSM 19808 might produce a nonribosomal nonapeptide resembling jagaricin, which is known to display hemolytic activity. Therefore, we deemed the *hml* locus worthy of further investigation.

### Gene inactivation of the *hml* BGC leads to loss of the β-hemolytic phenotype of *C. haemolyticum*

**Fig 2:**
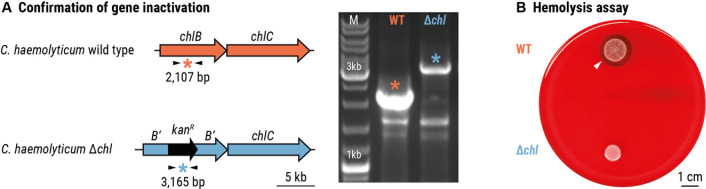
The products of the *hml*-encoded biosynthetic pathway mediate hemolytic activity of *C. haemolyticum*. (A) Verification of gene inactivation resulting from homologous recombination and incorporation of an antibiotic-resistant cassette. Left: primer binding sites (black arrows) and expected size of amplicons (indicated by *). Right: agarose gel containing amplicons obtained by PCR of genomic DNA of *C. haemolyticum* wild type (WT) and *C. haemolyticum* Δ*hml*. (B) Ten microliter culture spots on sheep blood agar of *C. haemolyticum* wild type, which is surrounded by a transparent zone of lysed erythrocytes (top), and *C. haemolyticum* Δ*hml*, which is not hemolytic (bottom). The white arrowhead indicates the boundary of the zone of hemolysis. Scale bar: 1 cm.

Due to the similarities between the *hml* and *jag* BGCs and given that jagaricin is a known hemolysin (37), we aimed to target the *hml* BGC for inactivation and test whether the hemolytic phenotype of *C. haemolyticum* is affected (10). Seeing as there are no reports of genetic modification of *C. haemolyticum*, we adopted a double-crossover strategy that was developed for the related Betaproteobacterium *J. agaricidamnosum* (36, 42). Using this strategy, the region encoding the C domain of module 4 in *hmlB* was disrupted in the genome of *C. haemolyticum* DSM 19808 (wild-type *C. haemolyticum*) to generate *C. haemolyticum* Δ*hml* (*C. haemolyticum* Δ*hml*::kan^R^) (Fig. 2A). Wild-type *C. haemolyticum* and *C. haemolyticum* Δ*hml* were inoculated on sheep blood agar, and after one day of incubation, their hemolytic activities were assessed. Wild-type *C. haemolyticum* forms a visible zone of hemolysis (Fig. 2B), whereas hemolytic activity is abolished in the case of *C. haemolyticum* Δ*hml* (Fig. 2B). This observation implicates an intact *hml* BGC in the hemolytic phenotype of *C. haemolyticum*.

### The *hml* BGC codes for the production of jagaricins, a family of cyclic lipodepsipeptides

**Fig 3:**
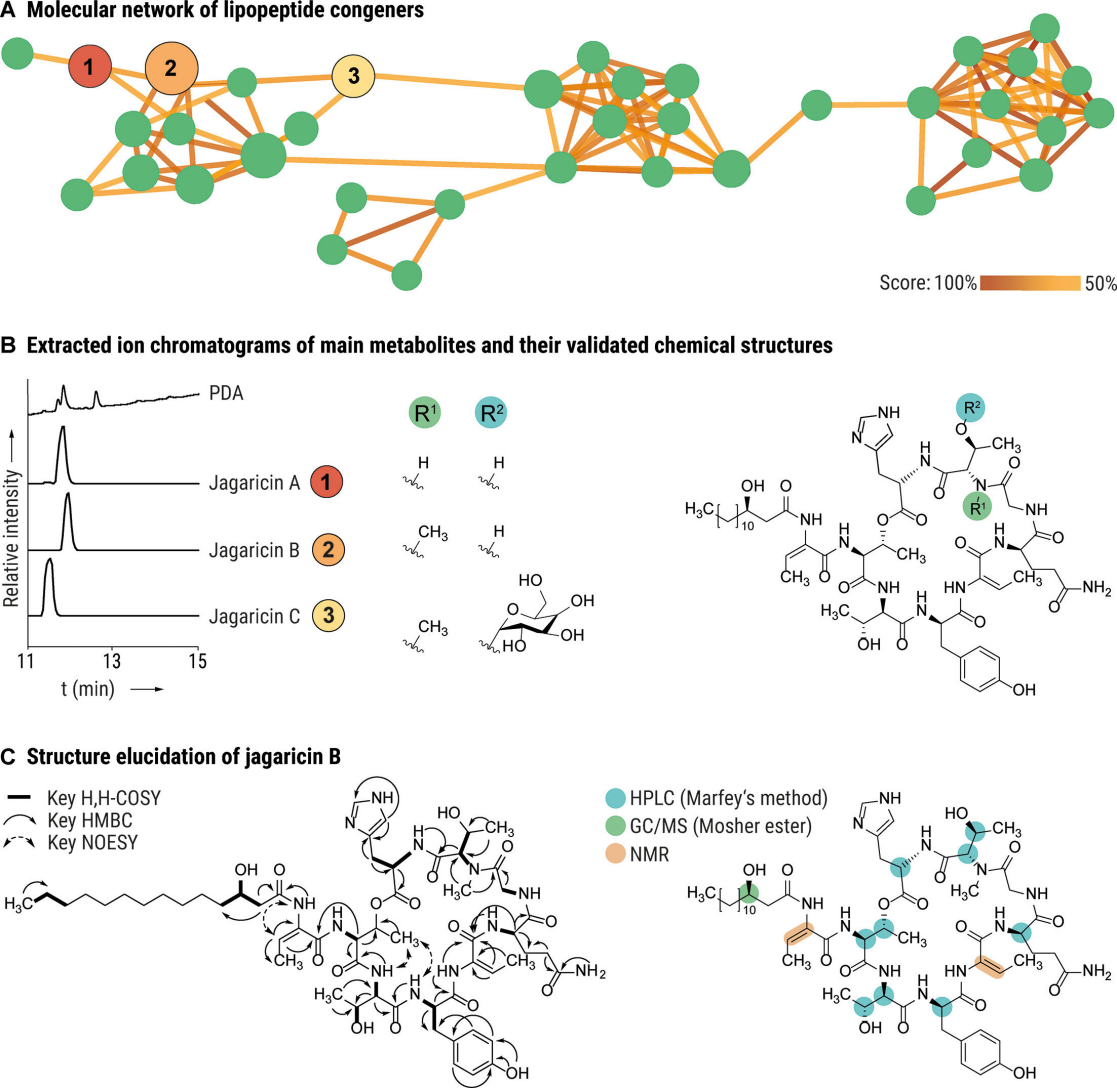
Discovery and elucidation of jagaricins. (A) Molecular network revealing the entire family of jagaricin congeners. Each node represents one compound; colored lines indicate structural similarity between two nodes based on MS² fragmentation. (B) Metabolic profile of *C. haemolyticum* DSM 19808 showing extracted ion chromatograms corresponding to the main congeners and their validated chemical structures (jagaricin A–C). (C) Structure elucidation of jagaricin B. Left: key 2D nuclear magnetic resonance connectivities; right: assignment of absolute configuration.

In light of the *hml* dependency of the hemolytic phenotype of *C. haemolyticum* DSM 19808, we sought to identify the cognate NRPS product(s) by metabolic profiling. To this end, we cultivated the strain in a variety of media and analyzed the culture extracts via high-performance liquid chromatography coupled with high-resolution mass spectrometry (HPLC/HRMS). We detected a number of species sharing an MS² fragmentation pattern that indicates a peptide sequence in line with our bioinformatic prediction. A molecular network based on MS² data was constructed to explore the quantity and structural similarity of putative congeners, revealing 37 related metabolites (Fig. 3A). As none of these compounds are produced by *C. haemolyticum* Δ*hml*, we conclude that the *hml* BGC codes for the biosynthesis of all congeners (Fig. S2).

The metabolic profiles showed that three main congeners were produced under the tested cultivation conditions (Fig. 3B). The following sum formulas were deduced from HRMS: **1**: C_56_H_85_N_12_O_16_ (*m*/*z* 1,181.6202 [*M* + H]^+^, calc. 1,181.6201); **2**: C_57_H_87_N_12_O_16_ (*m*/*z* 1,195.6359 [*M* + H]^+^, calc. 1,195.6358); and **3**: C_63_H_97_N_12_O_21_ (*m*/*z* 1,357.6906 [*M* + H]^+^, calc. 1,357.6886). We noted that the sum formula of **1** matches that of jagaricin. Indeed, comparison of MS^2^ spectra and HPLC retention time with a jagaricin standard confirmed that *C. haemolyticum* produces jagaricin (**1**) (Fig. S2). Therefore, we decided to collectively refer to the family of congeners produced by the *hml*-encoded biosynthetic machinery as the jagaricins and will refer to **1** as jagaricin A. Accordingly, congeners **2** and **3** were named jagaricin B and jagaricin C, respectively.

In order to obtain sufficient amounts of pure compounds for structural elucidation and bioactivity tests, a total of 15 L culture was extracted. Compound isolation using size-exclusion chromatography followed by preparative reverse-phase HPLC yielded 0.6 mg of jagaricin A (**1**), 5.6 mg of jagaricin B (**2**), and 1.5 mg of jagaricin C (**3**). We elucidated the structure of jagaricin B using nuclear magnetic resonance (NMR) spectroscopy and determined the absolute configuration by derivatization, followed by HPLC or gas chromatography coupled with mass spectrometry (GC/MS) analysis. These analyses revealed that jagaricin B is an *N*-methylated congener of jagaricin A (Fig. 3C; Fig. S4 and Table S3).

The deduced sum formula of jagaricin C deviates from that of jagaricin B by C_6_H_10_O_5_, and it has an MS² fragmentation pattern that differs by a neutral loss of a C_6_H_10_O_5_ fragment (Fig. S3). This signifies the presence of a sugar monomer in jagaricin C, which is likely incorporated by one of the glycosyl transferases encoded in the *hml* BGC. Based on MS²-fragmentation, we assume that *O*-glycosylation happens at the *N*-Me-Thr moiety, as is the case for the closely related hassallidin family (35). We isolated three additional congeners (jagaricin D–F) in sub-milligram amounts. These compounds putatively diverge by the length of the fatty acid chains and a replacement of histidine by tryptophan, as determined by NMR and MS² fragmentation (Fig. S7 to S9). Jagaricin B and C are identical to the previously described natural products Sch 20561 and Sch 20562, respectively, which are produced by human pathogenic *Aeromonas* species (43, 44) and putatively by mosquitocidal *Chromobacterium* sp. Panama, a strain that harbors a BGC similar to *hml* (Table S1) (15). A compound analogous to jagaricin B, named chromobactomycin, is also produced by the root-colonizing *Chromobacterium* sp. strain C61. Although jagaricin B and chromobactomycin have identical sum formulas, they differ in the order of the amino acids (45). In summary, we have identified the products of the *hml* BGC as jagaricins, a family of cyclic lipodepsipeptides.

### Jagaricins are potent hemolytic agents

**Fig 4:**
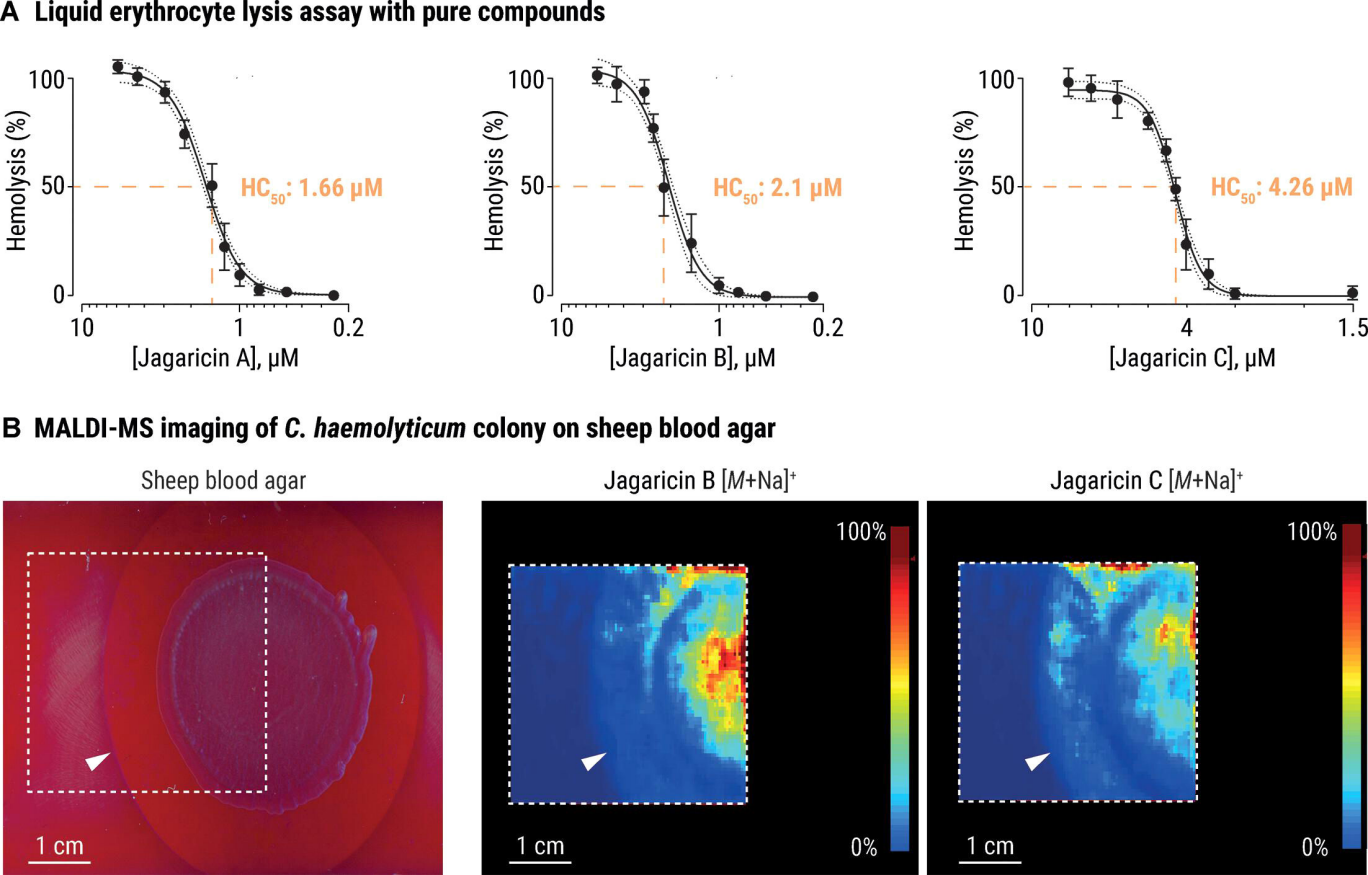
Jagaricins lyse sheep erythrocytes. (A) Hemolysis curves and 50% hemolytic concentration (HC_50_) values of jagaricin A (left), B (center), and C (right). Data points represent the averages derived from four independent experiments (*n* = 4 biological replicates, with three technical replicates each), and the error bars represent the standard deviation (±S.D.). The dotted lines indicate 95% confidence intervals. (B) Matrix-assisted laser desorption ionization mass spectrometry (MALDI-MS) imaging of a 10 µL culture spot of *C. haemolyticum* grown on sheep blood agar (left image). The distribution of jagaricin B (middle image) and jagaricin C (right image) in the colony, and hemolytic zone is indicated by a MALDI-MS imaging heat map, in which the color-coded score (0%–100%) indicates the relative abundance of the respective ions *m*/*z* = 1,217.6 Da (±0.5 Da, [*M* + Na]^+^) and *m*/*z* = 1,379.6 Da (±0.5 Da, [*M* + Na]^+^). Color code: blue, low abundance; red, high abundance of ion. The white arrowhead indicates the boundary of the zone of hemolysis, and the dashed square indicates the field of view for MALDI-MS imaging analysis. Scale bars: 1 cm.

We proceeded to test the three main cyclic lipodepsipeptides produced by *C. haemolyticum* DSM 19808 (jagaricin A–C) in a liquid erythrocyte lysis assay. Sheep erythrocytes were incubated with dilutions of each purified compound, and hemolysis was quantified by measuring the released hemoglobin in the supernatants. All three compounds display potent hemolytic activity in the low micromolar range (jagaricin A: 50% hemolytic concentration [HC_50_] = 1.66 µM, 95% confidence interval [CI] = 1.56–1.78 µM; jagaricin B: HC_50_ = 2.1 µM, CI = 1.96–2.23 µM; and jagaricin C: HC_50_ = 4.26 µM, CI = 4.19–4.33 µM; Fig. 4A), which is comparable with the previously reported potency of jagaricin A toward human erythrocytes (HC_50_ = 3.52 µM) (37). Our results establish that, like jagaricin A, jagaricin B and C are hemolysins.

To visualize the spatial distribution of jagaricins within and around a *C. haemolyticum* colony, we used matrix-assisted laser desorption ionization mass spectrometry (MALDI-MS) imaging. *C. haemolyticum* was cultured on sheep blood agar until a clear zone of hemolysis appeared surrounding the colony. The bacterial colony and zone were sprayed with a liquid matrix, and the imaging area was scanned. We detected jagaricin B and C within the colony and in the hemolytic plaque, while jagaricin A was absent (Fig. 4B). We attribute the β-hemolytic phenotype of *C. haemolyticum* to jagaricins based on the loss of this characteristic feature in a *hml* deletion mutant, together with the demonstrated hemolytic activity of purified metabolites and their *in situ* detection in a region of hemolysis.

### Jagaricins participate in behaviors associated with niche colonization

**Fig 5:**
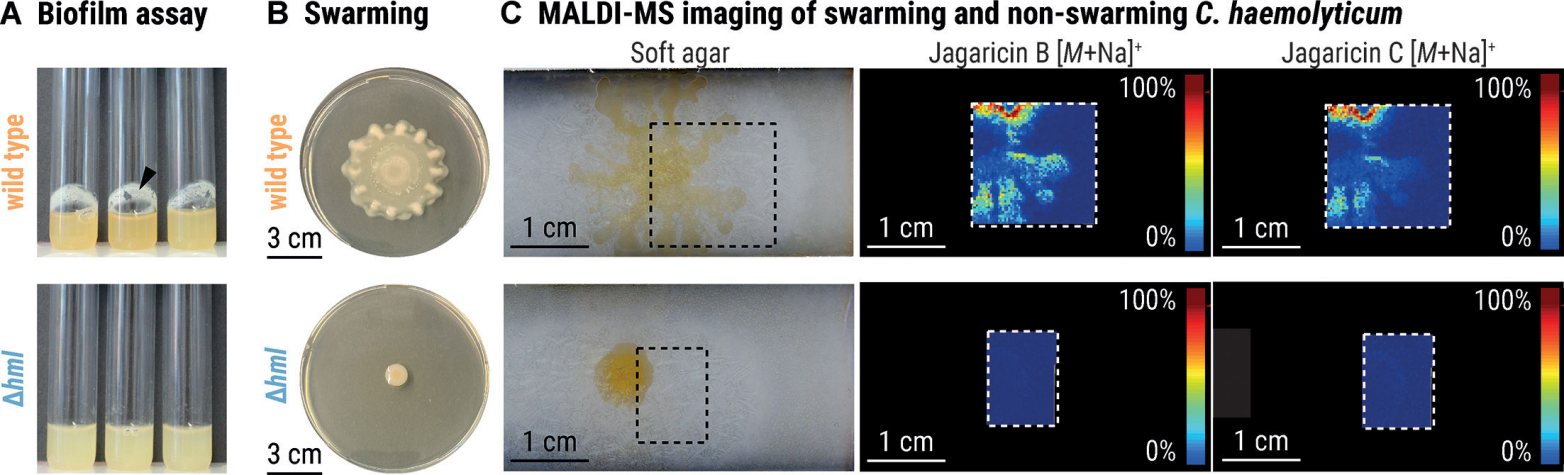
Jagaricins promote swarming and biofilm formation. (A) Biofilm assays of *C. haemolyticum* wild type (top) and *C. haemolyticum* Δ*hml* (bottom) grown in liquid cultures. The black arrowhead indicates the location of adhesive aggregates. (B) Swarming morphology of 10 µL culture spots of *C. haemolyticum* wild type (top) and *C. haemolyticum* Δ*hml* (bottom) on soft agar. (C) MALDI-MS imaging of 10 µL culture spots of *C. haemolyticum* wild type (top) and *C. haemolyticum* Δ*hml* (bottom) on soft agar demonstrates an accumulation of jagaricin B and C in the dendritic formations of the wild type and their absence in the deletion mutant. The color-coded score (0%–100%) indicates the relative abundance of the respective ions *m*/*z* = 1,217.6 Da (±0.5 Da, [*M* + Na]^+^) and *m*/*z* = 1,379.6 Da (± 0.5 Da, [*M* + Na]^+^). Color code: blue, low abundance; red, high abundance of ion. The dashed square indicates the field of view for MALDI-MS imaging analysis.

Since cyclic lipopeptides often function as biosurfactants, which can promote biofilm formation and swarming motility (46–51), we investigated whether jagaricins contribute to these important processes. To test whether the presence of an intact *hml* BGC is essential for biofilm formation, we cultivated the *C. haemolyticum* wild type and the *C. haemolyticum* Δ*hml* mutant in liquid cultures under slow agitation and visually monitored biofilm formation. After 5 days, wild-type *C. haemolyticum* produces a thick biofilm at the liquid-air interface, whereas *C. haemolyticum* Δ*hml* lacks the ability to form adhesive aggregates (Fig. 5A).

We cultured *C. haemolyticum* wild type and *C. haemolyticum* Δ*hml* on soft agar for 24 h to compare their swarming ability. *C. haemolyticum* wild type radiates outward in dendritic formations to colonize a large area of the agar surface, whereas *C. haemolyticum* Δ*hml* is unable to swarm (Fig. 5B). We inspected the *C. haemolyticum* wild-type colony with MALDI-MS imaging to visualize the distribution of jagaricin A–C during swarming. Of the three congeners, only jagaricin B and C accumulate in the dendritic formations of the swarming *C. haemolyticum* wild-type colony, suggesting preferential secretion at the periphery to improve motility. As expected, ions corresponding to jagaricin A–C were not detected in the case of *C. haemolyticum* Δ*hml* (Fig. 5C). The impaired ability of jagaricin-deficient *C. haemolyticum* to swarm and produce biofilms provides evidence that jagaricins are crucial for both processes, which may assist *C. haemolyticum* in colonizing new ecological niches (49, 51).

## DISCUSSION

Most ecosystems encompass a remarkable diversity of microbes and higher organisms that compete with one another for space and resources. One bacterial species that is evidently primed for such inter-organismal competition is *C. haemolyticum*, which is antagonistic toward coinhabitants of its freshwater niche, including bacteria (14), insects (13), and protists (15). *C. haemolyticum* is also equipped to transition to a pathogenic lifestyle, capable of infecting both humans (17–24) and other mammals (16). Despite mounting reports of serious diseases caused by *C. haemolyticum*, the molecular mechanisms underlying pathogenicity are largely unknown (28). In this study, we reveal a family of cyclic lipodepsipeptides that are responsible for the characteristic β-hemolytic phenotype of *C. haemolyticum*. Furthermore, we implicate these specialized metabolites in the swarming and biofilm-forming behavior of *C. haemolyticum*, which may impact niche colonization.

By means of comparative genomics, we discovered a cryptic NRPS-encoding BGC (*hml*) that is universally conserved in *C. haemolyticum* strains. We noted the putative BGC to bear striking similarity to that of jagaricin A, a cyclic lipodepsipeptide with reported hemolytic activity (37). Accordingly, we found that *C. haemolyticum* Δ*hml*, in which the *hml* BGC has been disrupted, lacks hemolytic activity. Using a combination of metabolic profiling, tandem mass spectrometry, and NMR, we went on to demonstrate that *C. haemolyticum* also produces jagaricin A, as well as many closely related congeners, and that these specialized metabolites originate from the *hml* BGC. The hemolytic potencies of the three major congeners jagaricin A–C were quantified (1.66–4.26 µM) and found to be comparable to that previously reported for jagaricin A (3.52 µM) (37), despite differences in some assay parameters (e.g., sheep vs human blood, measurement of absorbance at 405 vs 541 nm). The hemolytic activity of jagaricin A–C is likely due to their amphiphilic character, each consisting of a cyclic peptide moiety covalently linked to a lipid chain, which can cause disruption of membrane integrity (52). Perhaps surprisingly, given its status as an emerging opportunistic pathogen, the molecular basis of the hemolytic phenotype of *C. haemolyticum* was until now enigmatic. It should be mentioned that other hemolysins remain to be discovered within the *Chromobacterium* genus since several other species are weakly hemolytic, namely *Chromobacterium paludis* (53), *C. violaceum* (54), and *C. rhizoryzae* (30), although only the latter harbors an *hml*-like BGC (72% sequence similarity). Indeed, the hemolytic activity of *C. violaceum* is lost when proteins are inactivated through heat treatment or enzymatic digestion, indicating the likely involvement of a pore-forming protein (54).

Notably, jagaricin A was first reported as a major contributor to lesion formation in soft rot disease of the white button mushroom *Agaricus bisporus*, which is caused by *J. agaricidamnosum* (36). Jagaricin A was also found to display broad anti-eukaryotic effects, inhibiting numerous pathogenic fungi and social amoebae, as well as being toxic toward human cells, including erythrocytes (36, 37). Given that *J. agaricidamnosum* is not known to infect mammals, the biological relevance (if any) of the latter was unclear and appeared to be incidental to its membrane-disrupting properties (37). Yet, it is likely that the hemolytic properties of jagaricins come to the fore during *C. haemolyticum* infection. Our results, therefore, exemplify an intriguing case of context-dependent bioactivity, in which the same type of specialized metabolite is utilized by taxonomically distant bacteria (*J. agaricidamnosum* and *C. haemolyticum* belong to different orders within the class Betaproteobacteria) to manifest disease in distinct eukaryotic hosts. It is also possible that the anti-fungal and amoebicidal effects of jagaricin A (36, 37) provide both *J. agaricidamnosum* and *C. haemolyticum* with a competitive advantage in their respective environmental niches.

Regardless of structure, hemolysins are often associated with the pathogenic potential of their producers (55–57). One pertinent example is streptolysin S (SLS), a ribosomally synthesized and post-translationally modified peptide hemolysin that is an indispensable virulence factor of human pathogenic *Streptococcus pyogenes* (58). As is the case for many hemolysins, SLS destructs erythrocytes as a means to access nutrients, primarily iron (58–60). Iron is an essential element that plays catalytic, regulatory, and structural roles, as well as being instrumental as a virulence regulator (61). Perhaps jagaricins similarly participate in iron acquisition during the infection process of *C. haemolyticum*, thereby aiding survival in the host. Fittingly, the *hml* BGC harbors six genes (*hmlK–P*) that encode homologs of proteins involved in the transport of hemin (62, 63). Based on the resemblance of HmlK–P to the periplasmic binding-protein-dependent transport system for hemin (HemPRSTUV) of the animal pathogen *Yersinia enterocolitica* (64, 65), one could envision the following process occurring in *C. haemolyticum*: HmlK and HmlL proteins transport hemin across the outer membrane into the periplasm, where it is bound by HmlN. HmlM degrades hemin in the periplasm to liberate iron, which is transported into the cytoplasm across the inner membrane by HmlO and HmlP (64, 65).

Given that lipopeptides often act as biosurfactants that contribute to the swarming and biofilm-forming behavior of their producers, thereby facilitating movement along surfaces (66) and access to new niches (46–51), we probed a similar role for jagaricins. Indeed, *C. haemolyticum* Δ*hml* is deficient in swarming and biofilm formation. Our observations are in accordance with previous reports of cyclic lipopeptides positively influencing swarming and biofilm formation (50, 67). It is conceivable that both processes play a part in the survival of *C. haemolyticum* not only in the environment but also during the host stage. Swarming properties might equip *C. haemolyticum* with the ability to colonize animals if it encounters a susceptible host. Biofilm formation might enable subversion of innate immune defenses during the infection process (68). As previously mentioned, jagaricins could cause erythrocyte lysis, leading to iron release and accompanying enhanced bacterial proliferation. Notably, hemolysins can also be cytolytic for cells of the immune system, e.g., macrophages and neutrophils, which could further support immune evasion (58, 69). We thus propose that jagaricins could be considered as “dual-use” virulence factors, a concept that refers to the observation that factors that aid persistence in the environment can also promote virulence within a host (70), ultimately resulting in “accidental virulence” (71). This fits with the well-documented opportunistic nature of *C. haemolyticum* infection. Finally, it is worth mentioning the possible translational avenues arising from the data presented herein. The enzymatic cleavage of cyclic lipopeptides is commonly employed by competing bacteria in the environment (72, 73) and is thought of as a promising alternative approach to treat bacterial infections (74, 75). Thus, if a central role for jagaricins in virulence is ultimately substantiated, they may represent promising targets for the development of antivirulence therapeutics to combat *C. haemolyticum* infections.

Despite its increasing recognition as an opportunistic pathogen, little is currently known regarding the features that shape the pathobiology of *C. haemolyticum*. Similarly, there is a limited understanding of genetic determinants of niche colonization by *C. haemolyticum*. Here, we provide insight into both aspects by establishing that jagaricins act as chemical mediators that are not only behind the pronounced β-hemolytic phenotype of *C. haemolyticum* but are also crucial for biofilm formation and swarming motility. Our experimental evidence points to the possibility that *C. haemolyticum* employs dual-use specialized metabolites to survive and thrive in its environmental niche, while simultaneously being able to successfully switch to a pathogenic lifestyle once inside a human host. In illuminating the virulence mechanisms of *C. haemolyticum*, we lay the foundation for the development of antivirulence strategies against this serious emerging pathogen.

## MATERIALS AND METHODS

### Strains and growth conditions

*Chromobacterium haemolyticum* DSM 19808 (MDA0585^T^) was maintained on lysogeny broth (LB) (10 g/L tryptone, 5 g/L yeast extract, 5 g/L NaCl, 1 g/L glucose, and 15 g/L agar) at 37°C. For metabolic screening, *C. haemolyticum* was cultured in Medium 2 (2 g/L yeast extract, 0.45 g/L KH_2_PO_4_, 2.39 g/L Na_2_HPO_4_⋅12H_2_O, 1 g/L beef extract, 5 g/L NaCl, and 5 g/L peptone), Rich Medium (5 g/L yeast extract, 10 g/L peptone, 5 g/L casamino acids, 2 g/L beef extract, 5 g/L malt extract, 2 g/L glycerin, 1 g/L MgSO_4_⋅7H_2_O, and 0.05 g/L Tween 80), Jagaricin Production Medium (20 g/L d-mannitol, 20 g/L d-fructose, 10 g/L yeast extract, 5 g/L sodium l-glutamate, 6 mL/L trace element solution (4 g/L CaCl_2_⋅H_2_O, 1 g/L C_6_H_5_FeO_7_⋅H_2_O, 0.2 g/L MnSO_4_, 0.1 g/L ZnCl_2_, 0.04 g/L CuSO_4_⋅5H_2_O, 0.03 g/L CoCl_2_⋅6H_2_O, 0.03 g/L Na_2_MoO_4_⋅2H_2_O, and 0.1 g/L Na_2_B_4_O_7_⋅10H_2_O) (76), MGY (1.25 g/L yeast extract, 10 g/L glycerol, 7 g/L K_2_HPO_4_, 2 g/L KH_2_PO_4_, 0.588 g/L sodium citrate, 1 g/L (NH_4_)_2_SO_4_, and 0.1 g/L MgSO_4_), LB (10 g/L tryptone, 5 g/L yeast extract, 5 g/L NaCl, and 1 g/L glucose), or Potato Dextrose Broth (4 g/L potato extract and 20 g/L glucose). *Escherichia coli* TOP10 (Invitrogen, Thermo Fisher Scientific) and DNA-methyltransferase deficient *E. coli* ER2925 (New England Biolabs) were used for the construction and amplification of plasmids and cultured on solid LB medium at 37°C. *C. haemolyticum* mutants were selected on solid LB medium supplemented with 50 µg/mL kanamycin.

### Bioinformatic analysis

BGCs resembling the *hml* BGC were identified in bacterial genomes of the NCBI database using the EFI Genome Neighborhood Tool (77). The integrated BLASTp tool was used to compare products of the NRPS-encoding genes (GJA_RS00800–GJA_RS00815) to protein sequences of the UniProt database (BLAST sequences ≤ 100; *e*-value ≤ 10^–5^; and neighborhood window size 10). The results were analyzed using antiSMASH 6.0 (78). A synteny diagram (*e*-value ≤ 10^–3^; length ≥ 100; and identity value ≥35) was created with EasyFig 2.2.4 (79) and the integrated tBLASTx tool and was recolored with Adobe Illustrator.

### Generation of metabolic profiles

Bacterial cultures (100 mL) were grown for 3–4 days and then extracted with 100 mL ethyl acetate. The organic layers were dried over anhydrous Na_2_SO_4_, filtered using Macherey-Nagel MN 615 ¼ filter paper, and concentrated under reduced pressure. The crude extracts were dissolved in 1.3 mL methanol (MeOH), of which 25 µL was diluted with 25 µL MeOH before analysis by HPLC/HRMS using an Exactive Hybrid-Quadrupole-Orbitrap with electrospray ion source coupled to an Accela HPLC system (Thermo Fisher Scientific). For separation, a Betasil C18 column (2.1 × 150 mm, 3 µm, Thermo Fisher Scientific) was used with gradient elution (solvent A: H_2_O + 0.1% HCOOH; solvent B: MeCN + 0.1% HCOOH; gradient: 0–1 min 5% B; 1–16 min 5%–98% B; 16–20 min 98% B; and flow rate 0.2 mL/min).

### Tandem mass spectrometry

Samples were dissolved in MeOH, of which 3 µL was injected and analyzed using a QExactive Hybrid-Quadrupole-Orbitrap with electrospray ion source coupled to an Accela HPLC system (Thermo Fisher Scientific). For separation, an Accucore C18 column (2.1 × 100 mm, 2.6 µm, Thermo Fisher Scientific) was used with gradient elution (solvent A: H_2_O + 0.1% HCOOH; solvent B: MeCN + 0.1% HCOOH; gradient: 0–10 min 5%–98% B; 10–14 min 98% B; and flow rate 0.2 mL/min). MS^2^ spectra were generated for molecular network analysis by the top five analysis method (scan range *m*/*z* 400–1,500 and 25% normalized collision energy [HCD]).

### Mass spectrometry network analysis

A molecular network was created with MS^2^ spectra from *C. haemolyticum* culture extracts using Compound Discoverer 3.3. Only metabolites that yielded fragment ions were chosen for cluster generation. Two nodes were linked when the following parameters were fulfilled: Msn score ≥ 50%, minimal coverage ≥ 60%, and matching fragments ≥ 10. Node size is proportional to the peak area of the respective compound in the LC/MS spectrum. The colors of the links depend on the Msn Score (50%–100%) of the two linked metabolites. The network was subsequently cropped and recolored using Adobe Illustrator.

### Production and isolation of lipopeptides

*C. haemolyticum* was grown in 30 × 500 mL volumes of LB medium, totaling 15 L, for 3 days at 30°C without agitation. Each 500 mL culture was extracted with 300 mL ethyl acetate, and the combined organic layers were dried over anhydrous Na_2_SO_4_, filtered, and concentrated under reduced pressure. The crude extract was fractionated by size exclusion chromatography (Sephadex LH-20 column [440 × 35 mm] with MeOH as eluent). Fractions containing the desired compounds were further purified by preparative HPLC (Shimadzu LC-8a series, DAD detector) using a C-18 Grom-Saphir 110 column (250 × 20 mm, 5 µm) and gradient elution (solvent A: H_2_O + 0.01% trifluoroacetic acid [TFA]; solvent B: MeCN; gradient: 3 min 10% B; 10%–90% B in 35 min; and flow rate 10 mL/min). Fractions containing each of jagaricin A, B, and C were collected and then separately combined and lyophilized to yield 0.6, 5.6, and 1.5 mg purified compound, respectively. For physicochemical data, see the supplemental material.

### Nuclear magnetic resonance spectroscopy

NMR spectra were recorded in deuterated dimethyl sulfoxide (DMSO-*d_6_*) using a Bruker AVANCE III 600 MHz instrument equipped with Bruker Cryo Platform. Chemical shifts are reported in ppm relative to the solvent residual signal (^1^H: *δ* = 2.50 ppm and ^13^C: *δ* = 39.52 ppm) (80). The following abbreviations are used for multiplicities of resonance signals: s, singlet; d, doublet; t, triplet; q, quartet; and br, broad.

### Synthesis of *N*-methyl threonine reference compounds

Synthesis was performed as previously described (81, 82) with the following modifications. SOCl_2_ (910 µL) was added dropwise to dry MeOH (3.5 mL), and the solution was cooled to –10°C. l-Thr (350 mg) was then added slowly, and the reaction mixture was stirred for 14 h at ambient temperature before being diluted with 2 mL of a saturated aqueous NaHCO_3_ solution. The mixture was extracted with CH_2_Cl_2_ (four times); the combined organic extracts were dried over anhydrous Na_2_SO_4_, filtered using Macherey-Nagel MN 615 ¼ filter paper, and concentrated under reduced pressure, yielding the threonine methyl ester (l-Thr-O-Me). l-Thr-O-Me (110 mg) was dissolved in CH_2_Cl_2_ (11 mL), and 0.1 M aqueous trifluoroacetic acid (10 mL) was added before cooling the mixture to 0°C. Formaldehyde (37% aqueous solution, 69 µL) was added dropwise under vigorous stirring, which was continued for 8 h at ambient temperature before being neutralized with a saturated aqueous NaHCO_3_ solution. The mixture was extracted with CH_2_Cl_2_ (four times). The combined organic extracts were dried over anhydrous Na_2_SO_4_, filtered using Macherey-Nagel MN 615 ¼ filter paper, and concentrated under reduced pressure, yielding the corresponding oxazolidine derivative, which was used without further purification. Oxazolidine (8 mg) was dissolved in dry CH_2_Cl_2_ (0.8 mL), cooled to 0°C, and TFA (0.8 mL) was added, followed by the dropwise addition of triethyl silane (0.09 mL). The reaction mixture was stirred at ambient temperature for 24 h before being concentrated under reduced pressure. The pale yellow residue was resuspended in 1 M HCl and washed with petroleum ether. Hydrolysis in 6 M HCl (1 mL) under reflux for 16 h yielded 8 mg *N*-Me-l-Thr hydrochloride salt as a pale yellow foam. The same procedure was used to synthesize *N*-Me-l-*allo*-Thr, *N*-Me-d-Thr, and *N*-Me-d-*allo*-Thr from l-*allo*-Thr, l-Thr, and l-*allo*-Thr as starting material, respectively.

### Absolute configuration of jagaricin B amino acid residues

Hydrolysis of 1 mg jagaricin B was carried out in 1 mL of 6 M aqueous HCl solution supplemented with 0.05% phenol under reflux for 14 h. A 500 µL aliquot was concentrated under reduced pressure and then dissolved in 100 µL water and 50 µL 1 M aqueous NaHCO_3_ solution. Subsequently, 10 µL of a freshly prepared 10 mg/mL solution of 1-fluoro-2,4-dinitrophenyl-5-l-alanine-amide in acetone was added. The reaction mixture was stirred at 40°C for 1 h before being quenched by the addition of 25 µL 2 M aqueous HCl solution followed by 25 µL MeOH. Reference amino acids (1 mg) were treated identically, and all samples were diluted 1:4 with MeOH before analysis via analytical HPLC (Shimadzu LC-10 Avp series, DAD detector, LiChrospher 100 RP-18 endcapped column [250 × 4.6 mm, 5 µm], flow rate 1 mL/min, solvent A: H_2_O + 0.1% TFA, and solvent B: MeCN).

For the determination of the retention times of the synthetic references, two different gradients were applied: gradient A (0–4 min 25% B, 4–44 min 25%–40% B, and 44–45 min 40%–98% B) provided the retention times (min) of d-His 6.19, l-His 7.94, l-*allo*-Thr 13.19, d-*allo*-Thr 14.59, *N*-Me-d-Thr 15.25, d-Thr 17.18, *N*-Me-l-*allo*-Thr 18.65, *N*-Me-d-*allo*-Thr 19.94, l-Tyr 25.39, and d-Tyr 28.88. Gradient B (0–5 min 20% B, 5–35 min 20%–25% B, and 35–40 min 25%–98% B) yielded sufficient separation of d-Gln 22.81, l-Gln 23.34, l-Thr 23.87, *N*-Me-l-Thr 25.73, and d-*allo-*Thr 28.62.

The absolute configurations of the amino acid residues were assigned as l-His (7.97 min, gradient A), l-Thr (23.87 min, gradient B), *N*-Me-l-*allo*-Thr (18.81 min, gradient A), d-*allo-*Thr (14.68 min, gradient A and 28.58 min, gradient B), d-Gln (22.80 min, gradient B), and d-Tyr (29.19 min, gradient A).

Configuration of dehydrobutyrine moieties was deduced from ^1^H-NMR chemical shifts of the vinylic proton quartets (*δ =* 5.82 and 5.63), which correlated with the reported ^1^H-NMR data of Sch 20561 (**2**, *δ =* 5.81 and 5.70) and its glycosylated congener Sch 20562 (**3**, *δ =* 5.84 and 5.80) (43, 44). Synthesis of the *N*-acetylated and *O*-methylated (*E*)- and (*Z*)-isomers showed that the vinylic proton of the (*Z*)-isomer (*δ =* 6.48) is downshifted in comparison to the (*E*)-isomer (*δ =* 5.90) (44).

### Absolute configuration of jagaricin B β-hydroxy fatty acid residue

Derivatization of the free fatty acid was performed as described previously (83) with the following modifications. Hydrolyzed jagaricin B (0.5 mg) was dissolved in 200 µL MeOH, and 30 µL of a 2 M solution of (trimethylsilyl)diazomethane in hexane was added to the reaction mixture. After stirring at ambient temperature for 10 min, the mixture was dried under a nitrogen flow. The residue was dissolved in 400 µL of a 0.2 M solution of 4-dimethylaminopyridine in dichloromethane, and 15 µL of (*R*)-(–)-α-methoxy-α-(trifluoromethyl)phenylacetyl chloride was added. The reaction mixture was stirred at ambient temperature for 2 h before being dried under a nitrogen flow. During the reaction, the appearance of the solution changed from colorless to golden-yellow. Reference fatty acids β-(*R*)-hydroxymyristic acid (HMA) and β-(*R,S*)-HMA (1 mg) were treated identically, and all samples were dissolved in MeOH before analysis via GC/MS.

GC/MS analysis was performed on a Trace 1310 GC (Thermo Scientific) coupled with a TSQ 9000 electron impact-triple quad mass spectrometer (Thermo Scientific). A 4 mm SSL GC inlet glass liner with glass wool (P/N 453A1305) and a BPX5 capillary column (30 m, 0.25 mm inner diameter, 0.25 µm film) from Trajan (SGE) were used. The column was operated using helium carrier gas (0.6 mL/min) and split injection (split flow: 25 mL/min, split ratio: 15). The temperature of the injector was set to 250°C. The oven temperature was set to 40°C for 4 min and then increased to 120°C with a rate of 40°C/min, followed by a temperature increase to 250°C with a rate of 0.75°C/min. The MS transfer line was set to 300°C, and the ion source temperature was set to 200°C. Total ion current values were recorded in the mass range of 45–500 amu with an offset of 70 min and a dwell time of 0.2 s. The injection volume was 0.5 µL.

The references and the sample show peaks with an identical fragmentation pattern containing the main fragment ions *m*/*z* 189.0 and *m*/*z* 241.2 with a retention time of 142.94 min (β-(*R*)-HMA); 142.51 min and 142.92 min (β-(*R,S*)-HMA); and 142.94 min (sample). The fatty acid residue was, therefore, assigned as β-(*R*)-HMA.

### Generation of *C. haemolyticum* Δ*hml* using homologous recombination

The target for genetic inactivation was the second open reading frame of the putative NRPS BGC (GenBank accession of *hml* BGC: PQ303793, corresponding to *hmlB*). Two neighboring homologous regions (hr1 and hr2, ~940 bp) were amplified by PCR using the primer pair *nrps*_C4_hr1_fw/*nrps*_C4_hr1_rv and *nrps*_C4_hr2_fw/*nrps*_C4_hr2_rv, respectively (Table S2). Both DNA regions were extended by 15 bp to be complementary to the *Sma*I-linearized vector pGL42a (84) and by 8 bp overlapping a kanamycin resistance cassette (*kan*^R^). *Kan*^R^ was amplified using the primer pair *nrps*_*kan*^R^_fw/*nrps*_*kan*^R^_rv (Table S2) with an 8 bp overhang that binds hr1 and hr2, respectively. Following agarose gel electrophoresis, the resulting amplicons (hr1, hr2, and *kan^R^*) were purified using the Monarch DNA Gel Extraction Kit (New England Biolabs) and subsequently assembled using the In-Fusion HD Cloning Kit and the *Sma*I-linearized cloning vector pGL42a. The resulting plasmid (pKO*nrps*) was introduced into chemically competent TOP10 *E. coli* (Invitrogen, Thermo Fisher Scientific) following the manufacturer’s instructions. Transformants were selected on LB agar supplemented with 50 µg/mL kanamycin. Incubation proceeded at ambient temperature until colonies formed, which were cultivated overnight in 2 mL LB medium at 37°C with agitation. The vector pKO*nrps* was isolated using the Monarch Plasmid Miniprep Kit (New England Biolabs) and then verified using restriction digest followed by Sanger sequencing. Subsequently, pKO*nrps* was transferred to DNA methylase-deficient *E. coli* ER2925 before being isolated to obtain an unmethylated vector for transfer to *C. haemolyticum*. Competent *C. haemolyticum* cells were generated following a protocol previously developed for the Betaproteobacterium *Mycetohabitans rhizoxinica* (85); however, the growth medium was changed to LB. Unmethylated pKO*nrps* (20–100 ng) was added to 60 µL of cells prior to electroporation at 25 µF, 200 Ω, and 2.5–3 kV (Eppendorf Eporator, Eppendorf, Hamburg, Germany). The cells were recovered in 0.5 mL LB at 37°C for 3–4 h with orbital shaking and spread on LB agar supplemented with 50 µg/mL kanamycin. The resulting colonies were checked for the integration of *kan^R^* at the targeted region of the genome by colony PCR using Δ*hml* verification primers (Table S2). Once verified, *C. haemolyticum* Δ*hml* was conserved at –80°C in 50% glycerol cryogenic stocks.

### MALDI mass spectrometry imaging

To localize hemolytic natural products via imaging mass spectrometry, 10 µL of a *C. haemolyticum* or *C. haemolyticum* Δ*hml* overnight culture was pipetted onto tryptic soy agar supplemented with 5% sheep blood (Thermo Scientific Oxoid) and cultivated for 24 h at ambient temperature.

To localize natural products involved in swarming, 10 µL of a *C. haemolyticum* or *C. haemolyticum* Δ*hml* overnight culture was pipetted onto MGY soft agar (0.6% agar) and cultivated for 24 h at ambient temperature. Agar with bacterial growth was subsequently transferred to an indium tin oxide-coated slide and dried overnight at 37°C. Dried samples were sprayed with a 1:1 mixture of 20 mg/mL 2,5-dihydroxybenzoic acid and α-cyano-4-hydroxycinnamic acid in MeCN:MeOH:H_2_O (70:25:5) on an ImagePrep Station 2.0 (Bruker Dynamics) in 60 consecutive cycles (spraying for 1 s, incubating for 10 s, and drying for 10 s). After 30 cycles, samples were rotated by 180° to ensure even coverage.

The samples were analyzed with an UltrafleXtreme MALDI TOF/TOF (Bruker Daltonics) in positive reflector mode with flexControl 3.0. The flexControl method was calibrated with the Peptide Calibration Standard II (Bruker) before every measurement. Each sample was irradiated with 500 laser impulses in a 200 µm wide grid (10 random impulses per grid position), and ions in a mass range of *m*/*z* 160–2,160 were detected. The resulting spectra were processed with FlexImaging 4.1 or SCiLs Lab 2016b with baseline subtraction and analyzed for masses of interest (±0.5 Da) that were subsequently visualized. For graphical documentation, imaging heat maps were cropped, and the contrast was adjusted using Adobe Illustrator.

### Biofilm formation

LB medium (2 mL) was inoculated with *C. haemolyticum* DSM 19808 or *C. haemolyticum* Δ*hml* and incubated at 30°C with slow agitation (120 rpm). After 5 days, cultures were checked for the presence of biofilm on the glass wall.

### Erythrocyte lysis assay

A 1 mL volume of defibrinated sheep erythrocytes (Thermo Scientific Oxoid) was washed by resuspension in 50 mL phosphate-buffered saline (PBS; 137 mM NaCl, 2.7 mM KCl, 10 mM Na_2_ HPO_4_, and 2 mM KH_2_PO_4_; pH 7.4), followed by centrifugation at 300 × *g* for 10 min at 20°C. The supernatant, containing hemoglobin released from lysed erythrocytes during storage at 4°C, was gently decanted, and the erythrocytes were washed a second time. The intact erythrocytes remaining after washing were resuspended in PBS to give an approximately 3%–4% (vol/vol) suspension of erythrocytes in PBS. The erythrocyte suspension was adjusted with PBS so that the absorbance reading obtained when treated with the nonionic surfactant Triton X-100, which is commonly used as a positive control in hemolysis assays, was within the linear range at 405 nm (see below).

The erythrocyte lysis assay (86) was performed by adding 75 µL volumes of the erythrocyte suspension to wells of a 96-well U-bottom microtiter plate (PS, clear; Greiner Bio-One). Control and test solutions were added (75 µL) to erythrocyte-containing wells (1:1 dilution), resulting in a final erythrocyte concentration of approximately 1.5%–2% (vol/vol). The positive control (corresponding to 100% or complete hemolysis) solution was 0.2% Triton X-100 (0.1% [vol/vol] Triton X-100 final concentration). The vehicle control (corresponding to 0% or background hemolysis) solution was PBS containing 4% DMSO (2% [vol*/*vol] DMSO final concentration). Purified **1**, **2**, and **3** were dissolved in PBS containing 4% DMSO (2% [vol*/*vol] DMSO final concentration) to give test solutions covering a range of compound concentrations. All reactions were performed in triplicate (*n* = 3 technical replicates).

After incubation at 37°C for 1 h, the microtiter plate was centrifuged at 300 × *g* for 5 min at 20°C, and 100 µL volumes of the supernatants were transferred to a fresh microtiter plate using a pipette, ensuring not to disturb any sedimented intact erythrocytes. Hemoglobin release was quantified by measuring the absorption of the supernatants at 405 nm (A405) using a Varioskan LUX 3020-237 (Thermo Scientific) microplate reader with SkanIt Software for Microplate Readers (Research Edition, Product Version 7.0.0.50; Thermo Fisher Scientific).

The absorbance values from each set of technical replicates were averaged, and the extent of hemolysis (percentage hemolysis) caused by each compound concentration was calculated using the formula: ([average A405 of the treated samples at a given concentration – average A405 of vehicle-treated samples]/[average A405 of Triton-treated samples – average A405 of vehicle-treated samples]) × 100. The entire experiment was performed four times on different days (*n* = 4 biological replicates). The normalized percentage hemolysis values were plotted against the logarithmic value (log_10_) of the corresponding compound concentrations (µM) tested to determine the HC_50_ values using GraphPad Prism version 10.1.2 for Windows (GraphPad Software, Boston, MA, USA; www.graphpad.com). The data points represent the average, and the error bars represent the standard deviation (±S.D.) derived from four independent experiments (biological replicates; *n* = 4). The best-fit dose-response curve was determined using a log(inhibitor) vs response; variable slope (four parameters) fit. The fitting method was least squares regression, with no weighting and the bottom parameter fixed to zero. Confidence intervals were calculated (95%, profile likelihood) to visualize the extent to which the data define the true curve.

## Data Availability

All data generated or analyzed during this study are included in the article and the supplemental material. Primary NMR and MS data files are available on the Zenodo open access data server at the following link: https://doi.org/10.5281/zenodo.14796060.

## SUPPLEMENTAL MATERIAL

Supplemental material mbio.03605-24-s0001.docx Supplemental tables and figures.

ASM does not own the copyrights to Supplemental Material that may be linked to, or accessed through, an article. The authors have granted ASM a non-exclusive, world-wide license to publish the Supplemental Material files. Please contact the corresponding author directly for reuse.
